# Effect of Demographic Status and Lifestyle Habits on Glycaemic Levels in Apparently Healthy Subjects: A Cross-Sectional Study

**DOI:** 10.1155/2016/5240503

**Published:** 2016-11-23

**Authors:** Kasuni Nisansala Wijesena Walatara, Lohini Vijayendran Athiththan, Usha Kumari Hettiaratchi, Pradeep Rasika Perera

**Affiliations:** Department of Biochemistry, Faculty of Medical Sciences, University of Sri Jayewardenepura, Gangodawila, Sri Lanka

## Abstract

*Aim*. To identify the effects of sociodemographic status, family history, and lifestyle habits on fasting blood glucose (FBG) and fasting serum insulin (FSI) levels in apparently healthy subjects.* Methods*. Information was gathered using an interviewer-administered questionnaire from 227 apparently healthy nondiabetic subjects residing in a suburban area in Sri Lanka. Venous blood samples were collected after an overnight fast for FBG and FSI analysis. Correlations and differences were analyzed using SPSS (ver. 17) software.* Results*. The majority of the subjects were females, having secondary or tertiary education, monthly income ≥Rs. 25,000 (USD 175), and a positive family history of diabetes. Among the subjects, 10.1% were identified as prediabetics and the majority had familial diabetes with monthly income ≥Rs. 25,000 (USD 175). Subjects with high income had significantly higher mean FBG. In addition FBG had a significant correlation with age. Males and subjects with less than 6 hours/day sleeping duration at night had significantly higher FBG. Subjects with less vigorous physical activity and longer sitting duration had significantly higher FSI levels.* Conclusions*. Increasing age, higher income, positive familial history of diabetes, sedentary lifestyle, and short sleep at night have positive impact on glycaemic status in apparently healthy subjects.

## 1. Introduction

Diabetes mellitus is characterized by abnormal glucose metabolism and hyperglycaemia. It is a noncommunicable disease with significant global public health threat at present. Diabetes mellitus is attributed to ineffective use of insulin in the body [1]. Type 2 diabetes (T2D) is the most common form and mainly affects the adults; yet it is gradually becoming a disease of the younger generation as well [2]. T2D comprises 90% of people with diabetes worldwide [3].

The estimated global prevalence of diabetes among adults over 18 years of age was 9% in 2014 [3]. Furthermore, diabetes has become a significant health issue in Southeast Asia due to rapid development and global shift of the population. Moreover, due to rapidly rising rates of metabolic syndrome, there is an increase prevalence of early onset T2D [4, 5]. According to the International Diabetes Federation, the prevalence of diabetes in South Asia is estimated to be 8.3% in 2011 [2, 6]. Incidence of diabetes in Sri Lanka was found to be approximately 11% in 2010 where 36% were previously undiagnosed [4, 7, 8]. The incidence of prediabetes in Sri Lanka was estimated as 11.5% in 2005/2006 [9].

Diabetes has become one of the leading causes of death in the world [10]. The majority of the community with T2D remains oblivious of their condition for a long time. Although the exact cause of T2D is still not known, several risk factors are associated with T2D [2].

A national surveillance program carried out in India has identified that age and rapid urbanization are two significant risk factors for the increased prevalence of diabetes in India [11]. As insulin sensitivity alters with age, prevalence of T2D increases with age [12]. Among the modifiable risk factors, level of physical activity, obesity, and dietary habits are the major contributing factors for T2D. Evidence from prospective studies consistently supports the fact that males and females with normal weight and sufficient physical activity have a lesser risk of developing T2D when compared to obese, inactive groups [13]. Smoking and sleeping disturbances had been identified as risk factors for increase in insulin resistance (IR). Based on research findings IR is closely linked with chronic smoking [14]. Several prospective studies have shown different results on the risk of diabetes and pattern of sleeping. Rafalson et al. have found that short duration of sleep adds a relative risk for the development of impaired glycaemia [15].

Genetic predisposition is a strong determinant of T2D. Studies have stated that the prevalence of T2D varies in different ethnic origins from apparently similar environments [16]. A study carried out in United Kingdom had shown a higher prevalence of diabetes among South Asians, who were younger and less obese compared to native white Caucasians [9]. Also familial aggregation is an empirical factor to develop T2D as family members share a similar environment [16].

To our knowledge, no study has been carried out on the effect of risk factors such as sociodemographic status, lifestyle factors, level of physical activity, and family history on fasting blood sugar levels (FBG) and fasting serum insulin (FSI) among apparently healthy subjects without diabetes. Therefore, identifying the influence of all these risk factors on glycaemic levels in apparently healthy subjects (without diabetes) would be important as it can be used as a prealarming sign. Early identification would be of great importance to the individual to overcome the risk of getting diabetes. Hence, the aim of the present study was to identify the impact of sociodemographic status, family history, and lifestyle habits on FBG and FSI levels in apparently healthy subjects in Sri Lanka.

## 2. Methods

### 2.1. Study Design and Participants

A descriptive cross-sectional study was carried out at the Family Practice Centre, University of Sri Jayewardenepura, which is located in the suburbs of Colombo with multiple ethnicities. Nonprobability, convenience sampling was used and sample size was determined using the equation for estimating mean.

The study was approved by the Ethics Review Committee of University of Sri Jayewardenepura. After informing the study protocol, informed written consent was obtained from all participants. Apparently healthy 227 subjects who were not diagnosed as having diabetes (FBG < 6.9 mmol/L), between 20 and 70 years of age, who visited the centre were recruited from January to September 2013. Subjects who were pregnant, on steroidal drugs, having severe diseases or physical and cognitive impairments were excluded.

### 2.2. Data Collection

A pretest of the questionnaire was carried out among 10 subjects in the same study setting to validate the questionnaire (data not included in the study). The standardized, interviewer-administered questionnaire with open-ended and close-ended questions were used to collect data on sociodemographic characteristics, economical status, level of education, medical and family history, and behavioral and physical activity. Participants were given the opportunities to clarify their doubts in a familiar and comfortable language of the individual.

### 2.3. Blood Sample Collection and Biochemical Analysis

Overnight fasting blood samples were collected from each individual using empty sterile centrifuge tubes without anticoagulant according to the standard protocol. It was allowed to clot for 30–40 minutes at room temperature for serum separation. Simultaneously 150 *μ*L of blood was pipetted out into an Eppendorf tube with NaF for FBG analysis. Blood samples were centrifuged at 3600 rpm for 10 minutes at room temperature. Serum was separated and FBG analysis was done on the same day while serum for FSI analysis was stored at −20°C for batch assay. FBG was analyzed using glucose oxidase (GOD-PAP) kit (Biolabo reagent, Maizy) and FSI levels were measured with ELISA (DRG International, Inc., USA) method, respectively.

### 2.4. Definitions of Outcomes and Covariates

Subjects with FBG 3.9–5.6 mmol/L were classified as normal and those with FBG 5.6–6.9 mmol/L were classified as prediabetes according to IDF/WHO criteria [1]. Age was categorized as <35 years and ≥35 years. Monthly income was categorized as < and ≥Rs. 25,000 (USD 175). Education was categorized as none, primary, secondary, and tertiary [17]. Family history of diabetes (defined as having one or both parents with T2D) was taken into account in assessing a positive family history of diabetes.

Individuals who are smoking were classified based on whether the respondent was a nonsmoker (those who have never smoked 100 cigarettes during their lifetimes and not smoking at present), an ex-smoker (a person who had smoked at least 100 cigarettes during their lifetime but currently does not smoke), or a current smoker (a person who had smoked at least 100 cigarettes during their lifetime and reported smoking every day or some days at the time of the study) [18]. Frequency and extent of physical activity on a daily basis were recorded and grouped as vigorous (20 minutes of vigorous activity daily) or moderate (at least 30 minutes of moderate-intensity activity daily) and walking (at least 10 minutes per day) [19]. Sitting and sleeping were also taken into account [17]. Different cut-offs for duration of each activity were defined according to how they affect the blood glucose removal and insulin release [15, 20].

### 2.5. Statistical Analysis

All data were double-entered, cross-checked for consistency, and analyzed using the Statistical Package for Social Sciences version 17 (SPSS Inc., Chicago, IL, USA). Categorical variables were summarized by frequency and corresponding percentage, whereas continuous variables were summarized by mean and standard deviation. Pearson correlation was used to determine the correlation between continuous variables. The significance of differences between means for the categorical variables was identified by independent sample* t*-test.

## 3. Results

### 3.1. General Characteristics

General characteristics of the study population are presented in Table 1. Among 227 subjects, 40.1% were males and 59.9% were females. Mean age of the study population was 40.1 ± 13.7 years. Most of them lived in urban areas (65.6%) and had secondary or higher education (90.7%). Mainstream of the population (70.5%) had a monthly income of ≥Rs. 25,000 (USD 175). The incidence of smoking was negligible among the population and current smokers (7.9%) consisted of male subjects only. Greater part of the study population (97.4%) had performed either vigorous or moderate physical activities or walking on a daily basis.

### 3.2. Glycaemic and Insulinaemic Prevalence

**Figure 1:**
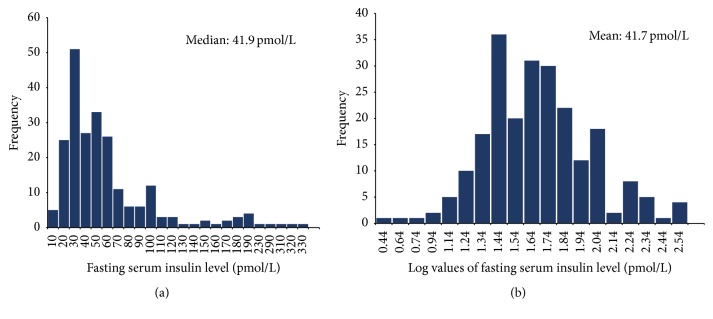
(a) Distribution of fasting serum insulin levels in the total study population (*n* = 227). (b) Distribution of natural log-scale of fasting serum insulin levels in the total study population (*n* = 227).

FBG levels were normally distributed among the study population with a mean ± 2SD of 4.6 ± 0.6 mmol/L. The distribution of FSI concentrations of the study population (Figure 1) was positively skewed, where the upper value for FSI in both males (13–224 pmol/L) and females (12–312 pmol/L) was above the normal range (16–172 pmol/L). The geometric mean FSI level for the total study population was 41.7 ± 2.1 pmol/L while 40.73 ± 2.18 pmol/L and 42.65 ± 2.08 pmol/L were for males and females, respectively.

A total of 23 subjects (10.1%) were detected as having prediabetes (FBG 5.6–6.9 mmol/L) with a mean FBG value of 6.1 ± 0.4 mmol/L and geometric mean FSI level of 63.1 ± 2.2 pmol/L. General characteristics of the subjects with prediabetes are shown in Table 2.

### 3.3. Influence of Sociodemographic Status and Family History on Glycaemia

There was a significant but a weak positive correlation between age and FBG (*p* = 0.000, *r* = 0.231) as shown in Table 3. Significantly higher FBG levels were found among males (*p* = 0.000) (Table 4). Among the subjects with prediabetes (Table 2), 52.2% were in the 30–49 years age group, 73.9% lived in urban areas, and 82.6% had a monthly income ≥Rs. 25,000 (USD 175). In addition among the subjects with prediabetes, significantly higher FSI levels were found in males (87.1 ± 2.1 pmol/L) compared to females (43.6 ± 2.1 pmol/L) as in Table 5. Among the subjects with prediabetes, mean FBG significantly (*p* = 0.024) differed with monthly income. Subjects with monthly income of ≥Rs. 25,000 (USD 175) were associated with higher mean FBG.

Among the study population, 47.6% of subjects and 65.2% subjects with prediabetes had a family history of diabetes. Significantly (*p* < 0.05) higher FBG and FSI levels were seen among the prediabetic subjects who had a positive family history of diabetes.

### 3.4. Influence of Physical Activity and Lifestyle Factors on Glycaemia

Physical activity and sedentary behavior have an effect on serum glucose levels. As shown in Table 3, duration of moderate physical activity and walking had a negative correlation with FBG which was not significant, but a significant negative correlation was observed for FSI with duration of walking whereas increased number of sitting hours showed a significant positive correlation (*p* < 0.05). In addition, subjects who did less vigorous physical activity (<2.3 hours) and longer sitting hours per day (>7.6 hours) had significantly high FSI levels (*p* < 0.05) (Table 4). Significantly high FBG levels were noted among prediabetic subjects who had prolonged sitting hours of >7.6 hours (*p* > 0.01) (Table 5).

Sleep loss and sleep disturbance also has an effect on glucose metabolism and development of IR. Sleeping hours at night had a negative correlation with FBG and subjects who slept <6 hours/day at night had significantly (*p* = 0.003) higher mean FBG levels (Table 4).

## 4. Discussion

T2D is a multifactorial disease with the involvement of multiple mechanisms. A combination of factors such as age, socioeconomic background, changes in levels of physical activity, dietary habits, lifestyle, and genetic susceptibility are known risk factors associated with occurrence of T2D. Numerous studies carried out in diabetic subjects have identified that socioeconomic level; low physical activity; and low diet quality are positive contributors to the development of diabetes [11–13]. However evidences for the effect of these factors in normal subjects are sparse. It is desirable to know how modifiable risk factors influence the risk of developing diabetes among apparently healthy people. Hence the aim of this study was to identify the impact of sociodemographic status, family history, and lifestyle habits on FBG and FSI levels in apparently healthy subjects. To the best of our knowledge, this is the first study to identify the influence of above factors on glycaemic levels in an apparently healthy population. The results showed that age, higher household income, family history of diabetes, short duration of moderate/vigorous physical activity, less walking hours, and short sleep duration contribute to an increase in FBG and FSI levels in the body.

Prediabetes is a preclinical asymptomatic state which has a high risk of progression to T2D. Numerous studies have found that the undiagnosed cases of IFG and IGT cause the progression to overt diabetes [21]. There were 10.1% subjects with undiagnosed prediabetes with high mean FBG and FSI level in our study. This denotes that undiagnosed prediabetes especially in apparently healthy population is a significant health issue in a community and this may be similar to other Asian countries. This highlights the requirement of timely and efficient screening programs, at national level and probably across the region, to identify individuals who have prediabetes and to prevent the progression to overt diabetes.

Familial aggregation is a strong risk factor for T2D and it is considered as one of the basic approaches in assessing potential risk of developing diabetes. Several studies have shown the positive contribution of the presence of familial aggregation in increasing the risk of diabetes [22–24] and Valdez et al. [23] have shown the graded association of familial diabetes. This study adds to the findings as 65.2% of subjects with prediabetes had a known family history. This study provides definitive evidence of the independent risk associated with family history of diabetes among apparently healthy subjects and prevalence of impaired glycaemic condition which ultimately has the potential to lead to T2D. This indicates the importance of proper screening at regular intervals of populations especially among subjects with a family history of diabetes.

It is evident that aging of population in a country significantly drives the epidemic of diabetes. Even though information on the relationship between age and FBG levels is relatively limited, increasing glycaemic levels with age in this study is in agreement with previous findings [12, 25]. Age had a very clear and significant positive correlation with FBG and also means FBG was higher in adult subjects over 35 years of age (*p* < 0.01). Surveillance data shows that apparently healthy, older adults (>45 years) are at high risk for both diabetes and prediabetes [26]. Our results are also in accordance with the above findings where the majority of the subjects with prediabetes (87%) were above 35 years of age and had a mean FBG of 6.0 ± 0.4 mmol/L. This suggests that increasing age in apparently healthy subjects, especially ≥35 years, could affect plasma glucose levels causing hyperglycemia. Thus it should be recommended that apparently healthy older adults should be screened in the clinical setting every 1–3 years for the early detection of T2D. Further studies have also stated that Asians are more prone to develop diabetes at an early stage compared to Caucasians.

The incidence rate of T2D is associated with low socioeconomic standards in developed countries whereas it shows an inverse relationship in developing countries [27]. Accordingly the majority of the subjects with prediabetes (82.6%) in this study had a monthly income ≥Rs. 25,000 (USD 175). Among the subjects with prediabetes, significantly higher mean FBG levels (*p* = 0.024) had a monthly income of ≥Rs. 25,000 (USD 175). Though some literatures from other countries have stated a mixed opinion on the role of income and prevalence of diabetes, in this study the association could be due to increased ability to purchase food in those who have a higher economic status leading to excess calorie intake. However, carrying out further studies would be helpful to understand the link between household income and T2D in order to provide more effective management and prevention of T2D.

Sleep loss and sleep disturbance have an effect in overall metabolic profile. Sleep is a restoration process required for homeostatic function, especially for glucose metabolism. Several prospective studies have identified U or J shaped relationship between sleep duration and T2D [15]. The present study found that subjects with night sleep duration <6 hours/day had significantly (*p* = 0.003) higher mean FBG. This association is evident to be clinically important as ones with short sleep duration have 30% increased risk of IR [28]. In contrast to our study findings, several studies have found that a longer sleep duration of longer than 8 hours/day leads to an increase in the risk of IFG by 60%. Both short and long sleep durations tend to increase the risk of T2D due to disordered glucose metabolism and increased cortisol levels which predispose to IR [15]. Our results reflect the clinical importance of sleeping 6–8 hours at night to reduce the risk of developing abnormal glucose metabolism. However, a future study with a larger but similar sample population would be able to assess the link between glycaemic levels and sleep quality, sleep pattern, and the presence of sleeping disorders in apparently healthy subjects.

Evidence from population based prospective studies indicates that longer sedentary hours and lesser moderate-to-vigorous-intensity physical activity have an independent influence in altering FBS in adults especially in subjects with unknown diabetes [13]. The majority of the study subjects in this study were involved with at least one type of physical activity. Negative correlations were identified with vigorous and moderate physical activities and walking activities which was not significant. Present findings demonstrate the importance of vigorous physical activity and its impact on FSI levels. In contrast, sedentary lifestyle with longer duration of sitting (>7.6 hours) has shown a significant positive influence on risk of developing diabetes. These findings are consistent with literature to support the effect of exercise in reducing the risk of hyperglycemia and hyperinsulinaemia. Further the results indicate that the benefit of exercise is also applicable to apparently healthy normoglycaemic subjects. Therefore, studies based on range of physical activity levels to identify the risk of T2D are needed to validate the above findings.

In the data analysis literacy status and cigarette smoking were not significantly associated with glycaemic and insulin levels. Yet, these factors are known risk factors for metabolic syndrome and known to be associated with altered glycaemia to a lesser extent [17]. However, the majority of our study sample consisted of nonsmokers (89%). Therefore, a larger sample size with smokers, in a similar population, might have a different finding to the present study.

The limitations of the present study include a convenience sample with cross-sectional study design. Secondly, assessment of lifestyle factors has a limitation as it was based on direct questioning of the participants only. When assessing family history of diabetes it would be useful if it could be stratified and expanded to include other first- and second-degree relatives. Also, self-reported data on sleep duration and level of physical activity was another limitation as it may result in overreporting of the duration. There are several notable strengths in our study: comparatively large sample size and participants were selected to represent the majority of the society and its applicability to the real world. Inclusion of a wide range of recognized sociodemographic and lifestyle risk factors allowed assessment of independent and combined influences on glycaemic states.

This study demonstrates that increasing age, higher household income, family history of diabetes, short sleep, and sedentary behavior contribute to increased fasting glycaemic and insulin levels in apparently healthy subjects as well. Undiagnosed prediabetics among apparently healthy subjects call for active screening of nondiabetic subjects in the community.

## Figures and Tables

**Table 1:** Characteristic of the participants.

Variables	*n*	%
*Demographic category*		
Gender		
Male	91	40.1
Female	136	59.9
Age		
18–29	69	30.4
30–49	91	40.1
50–70	67	29.5
Residential area		
Urban	149	65.6
Suburban	75	33.0
Rural	3	1.3
Level of education		
None	2	0.9
Primary	19	8.4
Secondary and above	206	90.7
Monthly income		
<Rs. 7,000 (<USD 49)	13	5.7
Rs. 7,000–25,000 (USD 49–175)	54	23.8
≥Rs. 25,000 (≥USD 175)	160	70.5
*Familial diabetes*		
Yes	108	47.6
No	119	52.4
*Life style patterns*		
Smoking		
Nonsmoker	202	89.0
Current smoker	18	7.9
Ex-smoker	7	3.1
*Physical activity and sleeping*		
Performance of physical activity		
Yes	221	97.4
No	6	2.6
Vigorous		
Yes	94	41.4
No	133	58.6
Duration of vigorous activities		
<2.3 h	153	67.4
>2.3 h	74	32.6
Moderate		
Yes	201	88.5
No	26	11.5
Duration of moderate activities		
<3.5 h	54	23.8
>3.5 h	173	76.2
Duration of sitting		
<7.6 h	32	18.9
>7.6 h	195	85.9
Duration of walking		
<3.5 h	30	13.2
>3.5 h	197	86.8
Sleeping at day		
Yes	181	79.7
No	46	20.3
Duration of sleeping at night		
<6 h	43	18.9
>6 h	184	81.1

General characteristics of the total study population (227) categorized according to the variables. *n* = number of subjects; %: percentage.

**Table 2:** Sociodemographic and life style characteristics among subjects with prediabetes.

Variables	*n*	%
*Demographic category *		
Gender		
Male	12	52.2
Female	11	47.8
Age		
18–29	1	4.3
30–49	12	52.2
50–70	10	43.5
Residential area		
Urban	17	73.9
Suburban	6	26.1
Level of education		
Primary	2	8.7
Secondary and above	21	91.3
Monthly income		
<Rs. 7,000 (<USD 49)	1	4.3
Rs. 7,000–25,000 (USD 49–175)	3	13.0
≥Rs. 25,000 (≥USD 175)	19	82.6
*Familial diabetes*		
Yes	15	65.2
No	8	34.8
*Life style patterns*		
Smoking		
Nonsmoker	19	82.6
Current smoker	4	17.4
*Physical activity and sleeping*		
Performance of physical activity		
Yes	23	100.0
Vigorous		
Yes	5	21.7
No	18	78.3
Duration of vigorous activities		
<2.3 h	20	87.0
>2.3 h	3	13.0
Moderate		
Yes	19	82.6
No	4	17.4
Duration of moderate activities		
<3.5 h	5	21.7
>3.5 h	18	78.3
Duration of sitting		
<7.6 h	2	8.7
>7.6 h	21	91.3
Duration of walking		
<3.5 h	4	8.7
>3.5 h	19	91.3
Sleeping at day		
Yes	17	73.9
No	6	26.1
Duration of sleeping at night		
<6 h	5	21.7
>6 h	18	78.3

General characteristics of the prediabetic population (23) categorized according to the variables. *n* = number of subjects; %: percentage.

**Table 3:** Correlation analysis of continuous variables with FBG and FSI.

	Biochemical parameter	
	FBG (mmol/L)	FSI (pmol/L)
*Demographic variable*		
Age	0.231^*∗∗*^	−0.009
*Physical Activity*		
Duration of vigorous activity	0.012	−0.112
Duration of moderate activity	−0.068	−0.076
Duration of walking	−0.040	−0.208^*∗∗*^
*Sitting and sleeping*		
Duration of sitting	−0.035	0.165^*∗*^
Duration of sleeping at night	−0.019	0.096
Duration of day time sleeping	−0.096	0.010

^*∗*^ *p* < 0.05 (2-tailed); ^*∗∗*^ *p* < 0.01 (2-tailed).

FBG: fasting blood glucose; FSI: fasting serum insulin.

**Table 4:** Comparison of FBG and FSI among different groups in categorical variables in total study population^†^.

Variables	*n*	Mean FBG (mmol/L) ± SD	*p* value	Mean FSI^*∗*^ (pmol/L) ± SD	*p* value
Gender					
Male	91	4.8 ± 0.6	*p* < 0.01	40.7 ± 2.2	*p* > 0.05
Female	136	4.5 ± 0.7		42.6 ± 2.1	
Monthly income					
<Rs. 25,000 (<USD 175)	67	4.6 ± 0.6	*p* > 0.05	36.3 ± 2.1	*p* > 0.05
≥Rs. 25,000 (≥USD 175)	160	4.7 ± 0.7		43.6 ± 2.1	
Familial diabetes					
Yes	108	4.7 ± 0.7	*p* > 0.05	44.7 ± 2.1	*p* > 0.05
No	119	4.6 ± 0.6		38.9 ± 2.1	
Smoking					
Yes	19	4.7 ± 0.6	*p* > 0.05	42.6 ± 1.9	*p* > 0.05
No	126	4.7 ± 0.6		35.4 ± 1.9	
Vigorous physical activity					
<2.3 h	153	4.7 ± 0.7	*p* > 0.05	46.7 ± 2.1	*p* < 0.01
>2.3 h	74	4.6 ± 0.6		33.1 ± 1.9	
Moderate physical activity					
<3.5 h	54	4.5 ± 0.6	*p* > 0.05	46.8 ± 2.1	*p* > 0.05
>3.5 h	173	4.7 ± 0.7		39.8 ± 2.1	
Sitting					
<7.6 h	32	4.5 ± 0.6	*p* > 0.05	30.9 ± 2.1	*p* < 0.01
>7.6 h	195	4.7 ± 0.7		43.7 ± 2.1	
Walking					
<3.5 h	30	4.7 ± 0.7	*p* > 0.05	48.9 ± 2.7	*p* > 0.05
>3.5 h	197	4.6 ± 0.7		40.7 ± 2.0	
Day time sleeping					
Yes	46	4.7 ± 0.7	*p* > 0.05	40.7 ± 2.2	*p* > 0.05
No	181	4.7 ± 0.7		43.7 ± 1.9	
Sleeping at night					
<6 h	43	4.9 ± 0.6	*p* < 0.01	38.9 ± 2.5	*p* > 0.05
>6 h	184	4.5 ± 0.7		42.7 ± 2.0	

^†^Independent sample *t*-test in 227 subjects.

^*∗*^Geometric mean of FSI was used (transformed from the natural logarithm).

FBG: fasting blood glucose; FSI: fasting serum insulin; SD: standard deviation.

**Table 5:** Comparison of FBG and FSI among different groups in categorical variables in prediabetic subjects^†^.

Variables	*n*	Mean FBG (mmol/L) ± SD	*p* value	Mean FSI^*∗*^ (pmol/L) ± SD	*p* value
Gender					
Male	12	6.0 ± 0.3	*p* > 0.05	87.1 ± 2.1	*p* < 0.05
Female	11	6.1 ± 0.5		43.6 ± 2.1	
Monthly income					
<Rs. 25,000 (<USD 175)	4	5.8 ± 0.2	*p* < 0.05	52.4 ± 2.5	*p* > 0.05
≥Rs. 25,000 (≥USD 175)	19	6.1 ± 0.4		64.5 ± 2.2	
Familial diabetes					
Yes	15	6.0 ± 0.4	*p* > 0.05	69.1 ± 1.9	*p* > 0.05
No	8	6.0 ± 0.4		50.1 ± 2.6	
Smoking					
Yes	4	6.1 ± 0.4	*p* > 0.05	63.1 ± 2.3	*p* > 0.05
No	19	6.0 ± 0.2		69.1 ± 1.8	
Vigorous physical activity					
<2.3 h	20	6.1 ± 0.4	*p* > 0.05	69.1 ± 2.2	*p* > 0.05
>2.3 h	3	5.9 ± 0.3		69.1 ± 1.5	
Moderate physical activity					
<3.5 h	5	5.7 ± 0.2	*p* > 0.05	72.4 ± 1.6	*p* > 0.05
>3.5 h	18	6.1 ± 0.4		60.3 ± 2.3	
Sitting					
<7.6 h	2	5.8 ± 0.0	*p* < 0.01	67.6 ± 1.8	*p* > 0.05
>7.6 h	21	6.1 ± 0.4		61.6 ± 2.2	
Walking					
<3.5 h	4	6.1 ± 0.5	*p* > 0.05	107.1 ± 2.1	*p* > 0.05
>3.5 h	19	6.0 ± 0.4		56.2 ± 2.1	
Day time sleeping					
Yes	6	6.1 ± 0.5	*p* > 0.05	67.6 ± 2.3	*p* > 0.05
No	17	6.0 ± 0.2		50.1 ± 2.1	
Sleeping at night					
<6 h	5	6.0 ± 0.2	*p* > 0.05	85.1 ± 2.5	*p* > 0.05
>6 h	18	6.1 ± 0.5		57.5 ± 2.1	

^†^Independent sample *t*-test in 23 subjects with prediabetes.

^*∗*^Geometric mean of FSI was used (transformed from the natural logarithm).

FBG: fasting blood glucose; FSI: fasting serum insulin; SD: standard deviation.
